# Expression signatures of *TP53 *mutations in serous ovarian cancers

**DOI:** 10.1186/1471-2407-10-237

**Published:** 2010-05-26

**Authors:** Marcus Q Bernardini, Tsukasa Baba, Paula S Lee, Jason C Barnett, Gregory P Sfakianos, Angeles Alvarez Secord, Susan K Murphy, Edwin Iversen, Jeffrey R Marks, Andrew Berchuck

**Affiliations:** 1 Department of Obstetrics and Gynecology, Division of Gynecologic Oncology, University of Toronto, 600 University Avenue, Toronto, Ontario M5G 2M9 Canada; 2 Department of Obstetrics and Gynecology, Division of Gynecologic Oncology, Duke University Medical Center, Durham, NC 27708, USA; 3 Department of Statistical Science, Duke University, Durham, NC 27708, USA; 4 Department of Surgery, Duke University Medical Center, Durham, NC 27708, USA

## Abstract

**Background:**

Mutations in the *TP53 *gene are extremely common and occur very early in the progression of serous ovarian cancers. Gene expression patterns that relate to mutational status may provide insight into the etiology and biology of the disease.

**Methods:**

The *TP53 *coding region was sequenced in 89 frozen serous ovarian cancers, 40 early stage (I/II) and 49 advanced stage (III/IV). Affymetrix U133A expression data was used to define gene expression patterns by mutation, type of mutation, and cancer stage.

**Results:**

Missense or chain terminating (null) mutations in *TP53 *were found in 59/89 (66%) ovarian cancers. Early stage cancers had a significantly higher rate of null mutations than late stage disease (38% vs. 8%, p < 0.03). In advanced stage cases, mutations were more prevalent in short term survivors than long term survivors (81% vs. 30%, p = 0.0004). Gene expression patterns had a robust ability to predict *TP53 *status within training data. By using early versus late stage disease for out of sample predictions, the signature derived from early stage cancers could accurately (86%) predict mutation status of late stage cancers.

**Conclusions:**

This represents the first attempt to define a genomic signature of *TP53 *mutation in ovarian cancer. Patterns of gene expression characteristic of *TP53 *mutation could be discerned and included several genes that are known p53 targets or have been described in the context of expression signatures of *TP53 *mutation in breast cancer.

## Background

The *TP53 *tumor suppressor gene encodes a transcription factor that plays a critical role in regulating cell cycle progression, DNA repair, and cell death. *TP53 *is the most frequently altered gene in human cancers and loss of functional p53 protein occurs in a majority of epithelial ovarian cancers. Ovarian cancers with serous histology account for about two-thirds of the incident disease and these cases usually present at an advanced stage leading to a very high mortality rate. In studies in which full gene sequencing has been performed, 60-70% of both early and advanced stage serous ovarian cancers harbor *TP53 *mutations [1,2]. In contrast, other histological subtypes of epithelial ovarian cancer that more commonly present at an early stage (endometrioid, clear cell, mucinous) have a much lower incidence of *TP53 *mutations [2]. Recently, *TP53 *mutations have been found in serous tubal intraepithelial carcinomas, usually in the context of "prophylactic" surgery in *BRCA1 *or *BRCA2 *mutations carriers. This suggests that *TP53 *mutation is an early event in the development of many serous cancers of the ovary, fallopian tube, and peritoneum [3-5].

It is possible that inactivation of the *TP53 *gene, or pathway, may be a requisite event in the development of serous cancers. In cancers that appear to retain the wild type coding sequence, p53 may be inactivated by other mechanisms (e.g., *TP53 *promoter or splicing alterations, *MDM2 *overexpression); or other genes that are regulated by *TP53 *may be altered obviating the need for direct inactivation of *TP53*. Alternatively, it is possible that the presence or absence of *TP53 *mutation in serous cancers may be one factor underlying the clinical heterogeneity of the disease with respect to stage, response to therapy, and outcome. Because p53 plays an important role in regulation of DNA damage response and repair as well as apoptosis, it has been postulated that inactivation of p53 in ovarian cancer is associated with resistance to cytotoxic therapy. However, a review of the extensive literature on p53 in ovarian cancer does not support a consistent relationship between *TP53 *mutation and/or overexpression and response to therapy or survival [6-8]. The absence of a clear association between *TP53 *alterations and outcome may reflect the complexity that underlies response to therapy or may be an indication that mutations in the coding sequence represent only a subset of the functional p53 alterations.

Since the p53 protein is a transcriptional activator, identification of an expression signature related to its activity has been pursued previously. Further, a number of genes have been demonstrated to be p53 inducible under various conditions and/or contain putative p53 response elements. However, since many of these experiments were performed either by transfection of wild type p53 or introduction of DNA damage, direct translation of this information to expression patterns in a given tissue that exists in a steady-state has been difficult. From breast cancer expression data, two separate groups found signatures related to the *TP53 *mutational status of the tumor [9,10]. While these signatures are somewhat consistent in the two studies, the complication in relating these expression patterns directly to p53 status is that mutations in breast cancer are much more likely to occur within a certain intrinsic subtype (basal), also defined by gene expression [11,12]. In the current study we attempt to derive, validate, and investigate the nature of a p53 expression signature in primary serous ovarian cancers and to relate those to signatures described in breast and other types of cancers.

## Methods

### Patient specimens

The specimens and relevant clinicopathological information were obtained with patient consent and used under protocols approved by the Duke University Institutional Review Board. Flash frozen specimens of 89 invasive serous ovarian cancers obtained at the time of initial surgery were utilized including 49 advanced stage (III/IV) and 40 early stage (I/II) cases (Table 1). All advanced stage cases and 7 of the early stage cases were obtained from the Duke Gynecologic Oncology Tumor bank and the remaining 33 early stage cases were obtained from either the Gynecologic Oncology Group (GOG) or Memorial Sloan Kettering Cancer Center. Treatment and outcome data were not available for the GOG cases.

**Table 1:** Clinical Characteristics of Study Population

	Early Stage (n = 40)	Advanced Stage (n = 49)
**Stage**	**Number (%)**	**Number (%)**
IA	7 (17.5%)	
IB	2 (5%)	
IC	7 (17.5%)	
IIA	1 (2.5%)	
IIB	6 (15%)	
IIC	17 (42.5%)	
III		39 (79.6%)
IV		10 (20.4%)
		
**Histological grade**		
Well differentiated	3 (7.5%)	2 (4.1%)
Moderately differentiated	9 (22.5%)	16 (32.6%)
Poorly differentiated	28 (70%)	31 (63.3%)

### Gene expression analysis

All expression data were previously generated from flash frozen tumor samples the Affymetrix U133A arrays and converted to MAS5 values [13]. The primary microarray data are available at http://data.genome.duke.edu/earlystageovc. We analyzed gene expression from formalin-fixed, paraffin-embedded samples in 38 of the advanced stage cancers using the Illumina Whole Genome DASL assay (cDNA-mediated annealing, selection, extension, and ligation) corresponding to 24,000 genes. Two slides containing 5 μm-thick sections of tumor were used for RNA extraction with the Ambion Recover All Total Nucleic Acid Isolation kit following the manufacturer's instructions. A total of 200 ng of RNA was then run on the Illumina Whole-Genome DASL Assay with the HumanRef-8 Bead Chip.

### *TP53 *gene sequencing

The mutational status of *TP53 *was examined by nucleotide sequencing of *TP53 *cDNA for all tumors in this study (*Tumor Mutation Information*, Additional File 1). Total RNA was isolated from flash frozen tissue specimens using RNA Stat-60 (Teltest; Friendswood, TX). cDNA was generated from one microgram of RNA using Roche (AMV) First Strand cDNA Synthesis Kit and random hexamer primers according to the manufacturer's recommendations (Roche Applied Science; Indianapolis, IN). PCR amplification was performed in a 25 μl reaction volume using 2.5 μl cDNA, 1.25 units Platinum Taq (Invitrogen; Carlsbad, CA), 0.2 mM each dNTP, 3 mM MgCl_2_, 5 μl Qiagen's Q Solution (Valencia, CA) and 0.4 μM each of forward primer F1 (5'-GAC ACG CTT CCC TGG ATT-3') and reverse primer R1 (5'-AGG GTT CAA AGA CCC AAA AC-3'). Cycling conditions were 94°C for 3 min, 35 cycles of 94°C for 30 sec, 61°C for 30 sec and 72°C for 2 min, followed by a final 5 min extension at 72°C. Amplicons were purified using Qiagen's MinElute PCR Purification Kit and sequenced in both directions using Big Dye Terminator Sequencing (Applied Biosystems; Foster City, CA). Sequencing primers included F1 listed above, F2 (5'-TTT TGC CAA CTG GCC AAG-3'), R2 (5'-GGT GGG AGG CTG TCA G-3'), and R3 (5'-GAG TCT TCC AGT GTG ATG ATG G-3'). The *TP53 *reference sequence NM_000546 was used for comparison to identify sequence variants present on the forward and reverse strands. Ambiguous results were resolved by sequencing of genomic DNA.

### Statistical Analysis

Data was imported into GeneSpringGx where all normalizations and analyses were performed. Initial filtering of probe sets was accomplished by applying an upper and lower threshold of expression (75-20000) of the raw MAS5 values before row and column centering for at least 15 of the 89 arrays. This resulted in 13,622 probes carried forward in common for all subsequent analyses. An unpaired T test was applied for each comparison with p values calculated asymptotically and corrected for multiple comparisons using the method of Benjamini-Hochberg [14]. Fold changes for the probes are expressed relative to the set of mutant p53 tumors in each case. A naïve Bayes (NB) classifier was then used for predictions employing the top genes found by the T test. The classifiers utilize the marginal frequencies of the outcome cases in the training set when making out-of-sample predictions. For in-sample predictions, a 3-fold validation was repeated 10 times to arrive at the final predictions. These algorithms were then used for predictions of out-of-sample sets, i.e. only early or only late stage cancers. Hierarchical clustering was performed on both probes and samples using a Pearson centered similarity measure. Probe lists used to make the naïve Bayes predictions can be found in Additional File 2.

For comparing gene expression data between the Affymetrix and Illumina DASL platforms, we tested for correlation by calculating a Pearson coefficient and the associated two-tailed P value.

## Results

The entire coding sequence of *TP53 *was analyzed in a set of 89 primary serous ovarian cancers comprised of 40 early stage (I/II) and 49 advanced stage (III/IV) cases (Table 1). The overall mutation rate was 66% in this series with a larger fraction of the early stage (78%) than late stage cancers (57%) harboring amino acid changing alterations (Table 2). The most common type of mutation led to single amino acid substitutions (68% of mutations) and the most common sites were in codons 220 and 273 (4 each) followed by 175 and 278 (3 each). Mutations that are predicted to result in a premature chain termination (referred to as "null") constituted 32% of the total. These null mutations were far more common in the early stage (38%) than late stage (8%) cancers (p = 0.003).

**Table 2:** Distribution of *TP53 *mutations by cancer stage and type of mutation.

	All Cancers	Stage I/II	Stage III/IV	**>7 yr Surv**.	<3 yr Surv
**Wild Type**	30	9	21	16	5
**Missense**	40	16	24	5	19
**Null**	19	15	4*	2	2
**Total Mutations**	59 (66.3%)	31 (77.5%)	28 (57%)	7 (30.4%)	21 (80.8%)**

* Null mutations as a fraction of total cases in early versus advanced cancers, p = 0.003

** Mutation rate in short versus long term survivors, p = 0.0004

While outcome data is not available for the majority of the early stage cancers, advanced stage cancers were from a previously published cohort used to derive an expression signature related to disease survival [15]. These cases were chosen to represent the extremes in outcome; one set was derived from women who survived less than 3 years after diagnosis and the other set from women with greater than 7 year survival from stage III/IV serous cancer. Mutations were much more prevalent in short term survivors (21/26, 81%) than long term survivors (7/23, 30%, p = 0.0004). Of the mutations in advanced stage cancers, only two from each group (short and long term survivors) were null with the remainder being missense changes. Therefore, the presence of a missense p53 mutation in this data set is associated with poor prognosis.

**Figure 1:**
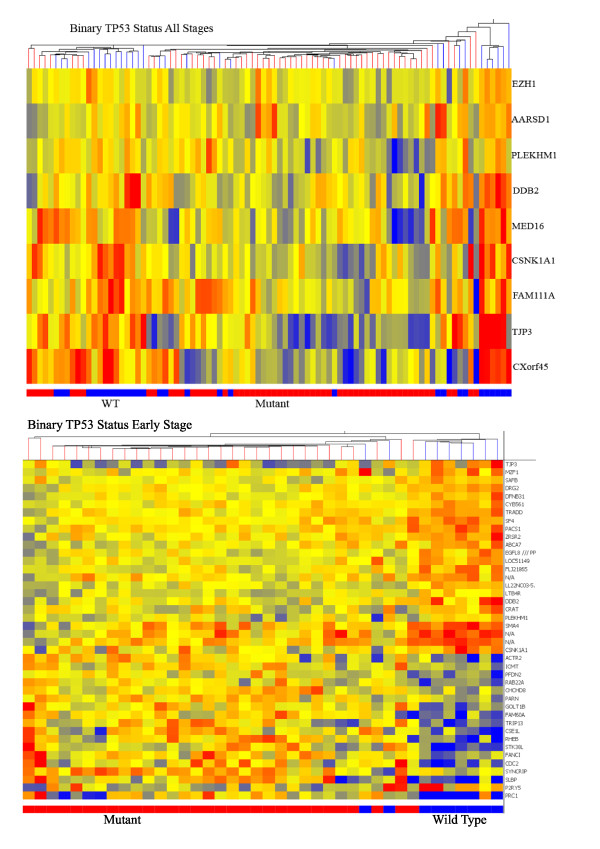
**Hierarchical clustering by both probes and samples**. A) All 89 cancers from the study were clustered using the 9 probes derived from a global T test of differential expression between mutant and wild type cancers. Actual p53 status is indicated on the bar below the heatmap (mutant = red and wild type = blue). Red indicates high level expression. B) The 40 early stage cancers were clustered by binary p53 status using the top 42 probe sets. Actual p53 status is indicated in the bar below the heatmap (mutant = red and wild type = blue).

Each of these tumors was previously analyzed for gene expression using Affymetrix U133A arrays [15,16]. From these data, we used p53 status to perform supervised classification. As a general approach, we first filtered the >22,000 probes sets by expression levels across all samples down to 13,622 probes for all subsequent analyses. An unpaired T test was then applied to the expression data using various categorical assignments of the ovarian cancers by stage and p53 status. The genes found to be most differentially expressed by the T tests were then used for prediction modeling. Predictions were made by naïve Bayes (NB) classifier with 3-fold validation repeated 10 times. The results of these T tests and predictions are shown in Table 3. Finally, examples of hierarchical clustering using these gene lists are shown in Figure 1 and in Additional File 3.

**Table 3:** Results of T test and prediction modeling.

Comparison	N	Misclassified	Accuracy	# Probes	P threshold	#Probes < 0.05
Mutant ***v ***WT (all stages)	89	11	87.6%	9	≤0.05	9
Missense ***v ***WT (all stages)	70	8	88.6%	21	≤0.11	1
Missense ***v ***Null (all stages)	59	8	86.4%	29	≤0.02	241
Mutant ***v ***WT (stage III/IV))	49	5	89.8%	68	≤0.14	0
Missense ***v ***WT (III/IV)	45	5	88.9%	17	≤0.07	1
Mutant ***v ***WT (stage I/II)	40	4	90%	42	≤0.36	0
Missense ***v ***WT (I/II)	25	1	96%	28	≤0.85	0
Null ***v ***WT (I/II)	24	2	91.7%	889	≤0.15	0

Cancers were categorized by p53 status; all mutant versus wild type or specific class of mutation (missense or null) versus wild type. We also sub-divided the cases by stage (I/II and III/IV) to further refine and explore the transcriptional signature related to p53 status. Analyzing the entire cohort of 89 cancers using binary p53 status (all mutant versus wild type), 9 probe sets were significant at the 0.05 level after Benjamini-Hochberg multiple testing correction. Using these probes, the mutational status of 77/89 (86.5%) cancers was correctly predicted. Of the 6 misclassified cancers that had sequencing verified mutations, 5 were missense mutants and half were early stage. Increasing the number of probes to 25 by decreasing the stringency of the T test (p = 0.15) resulted in a less robust predictor (13 cancers incorrectly assigned, 85.4%). The 9 probes represent 9 different genes (Table 4) and of these, one has been related previously to p53; *DDB2 *(Damage Specific DNA Binding Protein 2) [10,17]. Additional categories and results are given in Table 3. We used varying p value thresholds for probe selection due to the absence of probes significant at the 0.05 level. Nonetheless, predictions in the training sets for each comparison were reasonably accurate ranging from 86-96% correctly called samples. Since only 4 advanced stage cases harbored p53 null mutations, comparisons using this group were not performed.

**Table 4:** Genes in binary p53 signature.

Gene	Probe ID	p-value	Fold change*	Prior evidence for p53 association
***TJP3***	35148_at	7.96E-07	1.19	
***DDB2***	203409_at	1.44E-06	1.63	Yes
***AARSD1***	222064_s_at	8.10E-06	1.39	
***PLEKHM1***	212717_at	9.21E-06	1.39	
***CXorf45***	205583_s_at	1.82E-05	1.55	
***EZH1***	32259_at	2.50E-05	1.35	
***FAM111A***	218248_at	2.81E-05	1.27	
***MED16***	43544_at	3.09E-05	1.33	
***CSNK1A1***	208866_at	3.11E-05	1.26	

* Fold change is relative to mutant p53 containing tumors, i.e., all of these probes were elevated in wild type.

Besides the binary mutant versus wild type comparison for all stages, the only other comparison that yielded a set (> 1) of probes significant at the 0.05 level was between missense and null mutations. In this case, we set the threshold at p = 0.02 which resulted in 241 probe sets. The fact that most of the null mutations (15 of 19) were found in early stage cancers could be an indication that this comparison is confounded by disease stage.

Technical validation of the array data was accomplished using a series of 38 of the advanced stage cancers that were analyzed using the DASL platform from Illumina. Of the 9 genes in the binary p53 signature (Table 4), 5 (including the 4 top genes) demonstrated significant correlation between the two platforms (Additional File 4).

True validation of these signatures requires an independent set of serous ovarian cancers with matching array and sequencing data, however, no such data is available thus far. Using our data set alone, a number of relevant observations and tests can be made. This entails both comparisons of gene sets and predictions from early to advanced stage. Since all cancers in this study are of the serous type, it is reasonable to expect that the effects of p53 mutations should be similar in early and advanced stage. Therefore, to test the robustness of the p53 signatures, we compared probe lists derived separately from early and late stage cancers. For binary mutation status (wild type versus all mutant), the number of probes used to build the prediction models were 42 for early and 68 for advanced cancers. One probe (*CSNK1A1*) was in common between these lists (Table 3). Expanding to the top 100 probes for each condition yielded an additional common gene, *TP53 *itself. Finally, comparing the top 1000 probes gives 41 in common between early and late stage. The number of common probes does not exceed what might be found by chance (p = 0.36 for the intersection of the top 100 and p = 0.74 for the top 1000). The same type of analysis for wild type versus only missense mutations gave similar results. There are no probes in common from the lists used for the prediction models whereas 2 (*DDB2 *and *AARSD1*) of the top 100 and 46 of the top 1000 probes are coincident in the signatures from early and advanced cancers.

**Figure 2:**
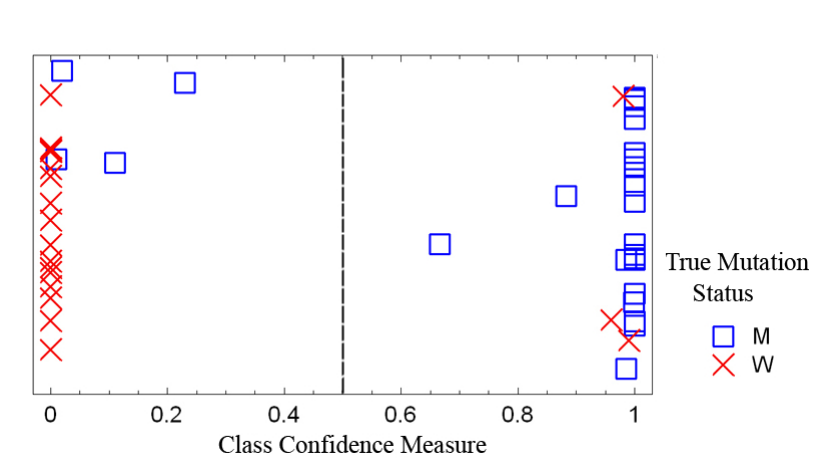
**Class prediction of binary p53 mutation status in advanced cancers using the algorithm derived from the binary status of early stage cancers**. The X axis is a measure of the confidence of the predictor for class assignment. Four mutant and three wild type tumors were mis-predicted. True mutational status is indicated by the symbols.

The small degree of overlap between probe sets suggests that predictions made using the early stage signature would have a low accuracy when applied to the late stage cancers and vice versa. Running the NB predictions in this way yielded accuracy values of 48%, 55.6%, and 47.5% for three of the four combinations (indicated in Table 5). However, when the model derived from early stage cancers based upon binary p53 status was applied to the group of late stage cancers, only 7/49 samples were mis-classified for an accuracy of 85.7% (Figure 2). This is appreciably better than chance and suggests that this signature may be the most biologically plausible. The genes (Table 6, 42 probes from 42 different transcription units) in this signature were compared to other published p53 signatures and transcriptionally related events. Three of the genes (*CDC2, DDB2*, and *TRIP13*) were found by Troester et al. in their p53 signature from breast cancer [10] and *DDB2 *is now a well established p53 target gene [17]. An additional three genes (*ACTR2*, *SAFB*, and *PRC1*) have been described as p53 transcriptional targets with varying levels and types of evidence [18-20]. As a comparison, of the 68 probes (representing 66 different transcribed regions) derived from the advanced stage cancers using binary p53 status, none have been identified in expression signatures or as p53 target genes.

**Table 5:** Intersection of p53 probes between early and advanced cancers.

	Mutant v Wild Type				Missense v Wild Type			
Stage	# probes*	Common	Top 100**	1000**	# probes	Common	Top 100	1000
Early	42				28			
		1	2	41		0	2	46
Advanced	68				17			

* Number of probes used for prediction modeling from Table 2

** The intersection of the top 100/1000 probe sets from early and advanced cancers

**Table 6:** Genes in early stage binary p53 signature.

Gene	Probe ID	p-value	Fold change*	Prior evidence for p53 association	Intersect with genes from Table 4
***DDB2***	203409_at	7.89E-05	1.56	Yes	Yes
***SLBP***	206052_s_at	1.05E-04	0.44		
***TTC38***	218272_at	1.41E-04	1.33		
***TRADD***	205641_s_at	1.87E-04	1.33		
***FLJ21865***	220349_s_at	1.93E-04	1.39		
***LTB4R***	216388_s_at	2.18E-04	1.28		
***CYB561***	207986_x_at	2.42E-04	1.26		
***GOLT1B***	218193_s_at	2.53E-04	0.62		
***SQSTM1***	220341_s_at	2.56E-04	1.37		
***PAPD4***	222282_at	2.68E-04	1.41		
***RHEB***	201453_x_at	3.11E-04	0.52		
***PACS1***	220557_s_at	3.60E-04	1.35		
***TRIP13***	204033_at	3.72E-04	0.59	Yes	
***P2RY5***	218589_at	3.76E-04	1.88		
***SYNCRIP***	209024_s_at	3.95E-04	0.64		
***ACTR2***	200728_at	4.33E-04	0.60	Yes	
***PLEKHM1***	212717_at	4.88E-04	1.37		Yes
***TJP3***	35148_at	4.88E-04	1.71		Yes
***RAB22A***	218360_at	5.07E-04	0.68		
***PARN***	203905_at	5.51E-04	0.71		
***DFNB31***	221887_s_at	5.59E-04	1.29		
***CHCHD8***	220647_s_at	6.41E-04	0.70		
***EGFL8***	208469_s_at	6.52E-04	1.34		
***ZRSR2***	208174_x_at	7.50E-04	1.38		
***DRG2***	203268_s_at	7.51E-04	1.27		
***ICMT***	201611_s_at	8.01E-04	0.69		
***SMA4***	206565_x_at	8.06E-04	1.59		
***GPC6***	215387_x_at	8.60E-04	1.62		
***CRAT***	209522_s_at	8.84E-04	1.43		
***PRC1***	218009_s_at	8.86E-04	0.47	Yes	
***CSE1L***	210766_s_at	9.46E-04	0.58		
***17p12 EST***	216751_at	9.58E-04	1.46		
***MZF1***	40569_at	9.64E-04	1.49		
***FANCI***	213007_at	9.68E-04	0.60		
***ABCA7***	219577_s_at	9.78E-04	1.36		
***SAFB***	201748_s_at	9.79E-04	1.25	Yes	
***PFDN2***	218336_at	9.87E-04	0.71		
***CDC2***	203213_at	9.96E-04	0.54	Yes	
***FAM60A***	220147_s_at	0.001024	0.62		
***STK38L***	212572_at	0.001033	0.58		
***SF4***	209547_s_at	0.001101	1.29		
***CSNK1A1***	206562_s_at	0.001107	1.54		Yes

* Mean fold change is relative to mutant p53 containing tumors

## Discussion

Mutations in the p53 gene are extremely common in epithelial ovarian cancers, particularly those with serous histology. Recent analyses of prophylactic salpingo-oophorectomy specimens from *BRCA1 *carriers show that the acquisition of these mutations may be a very early event in the development of many cases [3,21,22]. Given the prevalence and potentially pivotal nature of these mutations in the etiology of the disease, we sought to define transcriptional patterns associated with p53 mutational status in a series of early and advanced serous ovarian cancers. Since all cancers are of the serous histology, this should serve to reduce false associations that might arise in a study of mixed histologic types where the prevalence of p53 mutations varies, i.e., patterns of expression that are related to histology rather than the disruption of p53 activity.

In a total of 89 cancers, we sequenced the entire p53 coding region and performed expression analysis using Affymetrix U133A arrays. We categorized the cancers by stage (40 stage I/II and 49 stage III/IV cancers). *TP53 *mutational status was considered as a binary (mutations that alter the coding sequence versus wild type) and ternary (premature chain terminating mutation, missense, and wild type) variable to include possible differences in biology between the types of mutation. The overall mutation rate of 66% in this series is comparable to previous studies that have sequenced the full coding sequence of p53 in ovarian cancers [1,2,8,23-25]. Analyzed by stage, mutations were more prevalent in the early stage cancers (77% versus 63%) and the early stage cancers had a significantly higher incidence of null mutations (38% versus 8%). This is consistent with the report by Leitao et al. that focused on early stage ovarian cancers [2]. In 21 early stage serous cancers, 14 (67%) harbored mutations and of these, 9 were chain terminating. Therefore, while the mutation prevalence between early and late stage serous cancers is comparable, the rate of null mutations is significantly different (p < 0.001 for the combined data from Leitao et al. and our current study). Since mutations are not likely to be lost during disease progression, the high frequency of null mutations in the early stage cancers may be an indication of differences in molecular pathogenesis relative to advanced cancers. Whether this is also related to the more favorable clinical outcome of early stage cancers is unknown.

We broadly categorized p53 mutations as either premature chain terminating (null) or missense in our gene expression analysis. Beyond the incidence of these mutations in early and advanced ovarian cancers, there is a significant amount of evidence indicating that increased stability of missense p53 mutant proteins and subsequent accumulation in the nucleus leads to various transcriptional and biological consequences over and above what is seen when the protein is absent (as is the case with most chain terminating mutations) [26-28].

Several studies correlated the type of p53 mutation with clinical variables of epithelial ovarian cancers. Sood et al. examined the presence of distant metastases (parenchyma of the liver or spleen or extra-abdominal) in relation to p53 mutations [8]. The most significant finding was that distant metastasis was 8-fold more common in patients with cancers that carried null mutations compared to those with either missense mutations or wild type p53. In addition, Shahin et al. found that ovarian cancers with null mutations and functionally null tumors (based upon lack of p53 immunostaining) had the worst prognosis [25]. Therefore, two independent studies found that null mutations, at least in advanced stage cancers, seem to confer a more aggressive biology related to metastasis and outcome. While our current study was not specifically designed to test disease outcome, the advanced stage cancers were derived from women who survived less than 3 years after initial diagnosis (n = 26) and those who survived greater than 7 years (n = 23) [16]. Comparing mutation status to outcome in our advanced stage cases, the overall prevalence of mutations was significantly higher in the short term survivors (81% versus 30%) and both the long and short term survivor groups contained 2 cancers with null mutations. Therefore, while mutation status does correlate with survival in our series of advanced stage cancers, the primary difference is in the rate of missense mutations. A consistent and reproducible association between p53 mutations and ovarian cancer outcome remains elusive.

The primary goal of the current study was to determine whether a gene expression signature related to p53 status exists in ovarian cancer and could provide molecular insight into the disease. To our knowledge, this is the first exercise of its type applied to epithelial ovarian cancer but similar studies have been published for other cancers. Further, there is a large literature on transcriptional targets of p53 activity. Therefore, our study can be placed judiciously into this broader context. In the present study, using standard analytic approaches, gene lists of varying significance were derived between groups of tumors based on p53 status. A comparison of *TP53 *wild type versus mutant ovarian cancer of all stages yielded a set of 9 differentially expressed genes (at the p < 0.05 level using a relatively stringent false discovery criterion). Within sample predictions using these 9 genes yields an accuracy of 86.5%. Of these 9 genes, only *DDB2 *has other experimental evidence indicating that it is a *bona fide *p53 target. Transcription of *DDB2 *is activated by human p53 protein, the promoter element interacts with p53, it is part of the xeroderma pigmentosum complementation group E (XPE), and is involved in DNA damage recognition and repair [17,29-32]. Further, this is one of the 52 genes described by Troester et al. that discriminates breast cancers with p53 mutations. This gene appears to be a highly plausible candidate for membership in a cancer associated p53 gene expression signature.

With no appropriate validation set yet available, the strength of this overall predictor of *TP53 *mutation status cannot be evaluated directly. However, we do present a series of other predictors that can be tested within our data set and related to other published p53 signatures. In particular, the most robust approach within the constraints of the current study is to compare signatures derived in early versus late stage disease. Since all cancers were of serous histology, with relatively equal distribution by grade, the biologic consequences of a p53 mutation should be similar. Therefore, a signature derived on early or late stage cancers and then applied to the remaining cohort for "validation" should provide insight into the strength of the predictor. We observed minimal overlap in the gene sets that constitute these individual predictors (Table 5) presaging a failure to validate. Indeed, for three out of four of these predictors, accuracy in the validation sets hovered around 50%. However, testing the gene set derived from early stage cancers that were categorized by binary p53 status on the set of advanced cancers resulted in predictions with 86% accuracy. Examination of the genes constituting this predictor provides further support that this is a biologically meaningful result (Table 6). Of the 42 genes in this list, 6 (*DDB2, CDC2, TRIP13, ACTR2, PCR1*, and *SAFB*) have either been described in a p53 related breast cancer signature or implicated as p53 target genes by other means. For comparison, there is a single gene (*MYBL2*) in common between the two published p53 breast cancer signatures and no genes in common between either of the breast signatures and a similarly derived colon cancer signature [9,10,33].

The primary confounder in discerning a clear expression signature of *TP53 *mutation may be the fundamental importance of this pathway in the development of serous ovarian cancers. In the current series, two thirds of the cancers harbored p53 mutations. The question is whether the remaining third have other alterations that result in inactivation of downstream p53 functions. Numerous mechanisms have been described that may impact on the activity of p53 and there is no widely accepted metric for evaluating this parameter, particularly from a frozen tissue sample. Gene expression patterns indicative of mutational status in ovarian cancers are discernible, but they are not highly predictive in out of sample validation with the exception of a signature developed specifically on early stage cancers. However, a number of genes within this signature have a high degree of biologic plausibility. The confirmation of several of these genes in a p53 related breast cancer signature suggests that a core set of expression markers could be developed to assess p53 activity in multiple cancer types.

## Conclusions

Patterns of gene expression in serous ovarian cancers that are characteristic of *TP53 *mutation can be discerned and include several genes that are known p53 targets or have been described in the context of expression signatures of *TP53 *mutation in breast cancer. The high frequency of mutations in these cancers, particularly in high grade disease, suggests that functional inactivation of this pathway may be an obligate event.

## Competing interests

The authors declare that they have no competing interests.

## Authors' contributions

MQB performed and analyzed the sequencing of ovarian cancers for *TP53 *mutations, participated in the generation of gene expression signatures, and in writing the manuscript. TB, GS, and AS participated in the generation of gene expression signatures. PSL and JCB performed and analyzed *TP53 *sequencing of a subset of the cancers. SKM supervised the laboratory component of the project and participated in conception of the overall study design. EI participated in the generation of gene expression signatures. JRM participated in the generation of gene expression signatures, annotated gene lists, in the overall study design, and in preparation of the manuscript. AB conceived the study design and contributed to the drafting and editing of the manuscript. All authors have read and approved the final version of the manuscript.

## Pre-publication history

The pre-publication history for this paper can be accessed here:

http://www.biomedcentral.com/1471-2407/10/237/prepub

## Supplementary Material

Additional File 1 **Tumor Mutation Information**. An Excel spreadsheet containing the tumors analyzed with mutation information. Click here for file

Additional File 2 **Probe Lists**. An Excel spreadsheet containing the probe lists used for each of the analyses described in the manuscript. Click here for file

Additional File 3 **Additional Heatmaps not in the Manuscript**. A PDF file containing supervised clustering of cancers by p53 mutation status not shown in the published manuscript. Click here for file

Additional File 4 **Affymetrix DASL Correlations**. The comparison of expression values for selected genes for a subset of cancers between Affymetrix data derived from frozen material and DASL data derived from the same cancers that were formalin-fixed and paraffin embedded. Click here for file
