# Supplementary material for: Expression signatures of TP53 mutations in serous ovarian cancers
Source: BMC Cancer. 2010 May 26;10:237. doi: 10.1186/1471-2407-10-237 (PMC2893110; doi:10.1186/1471-2407-10-237)
Supplement: Additional File 3 — Additional Heatmaps not in the Manuscript. A PDF file containing supervised clustering of cancers by p53 mutation status not shown in the published manuscript. [file 1471-2407-10-237-S3.PDF]

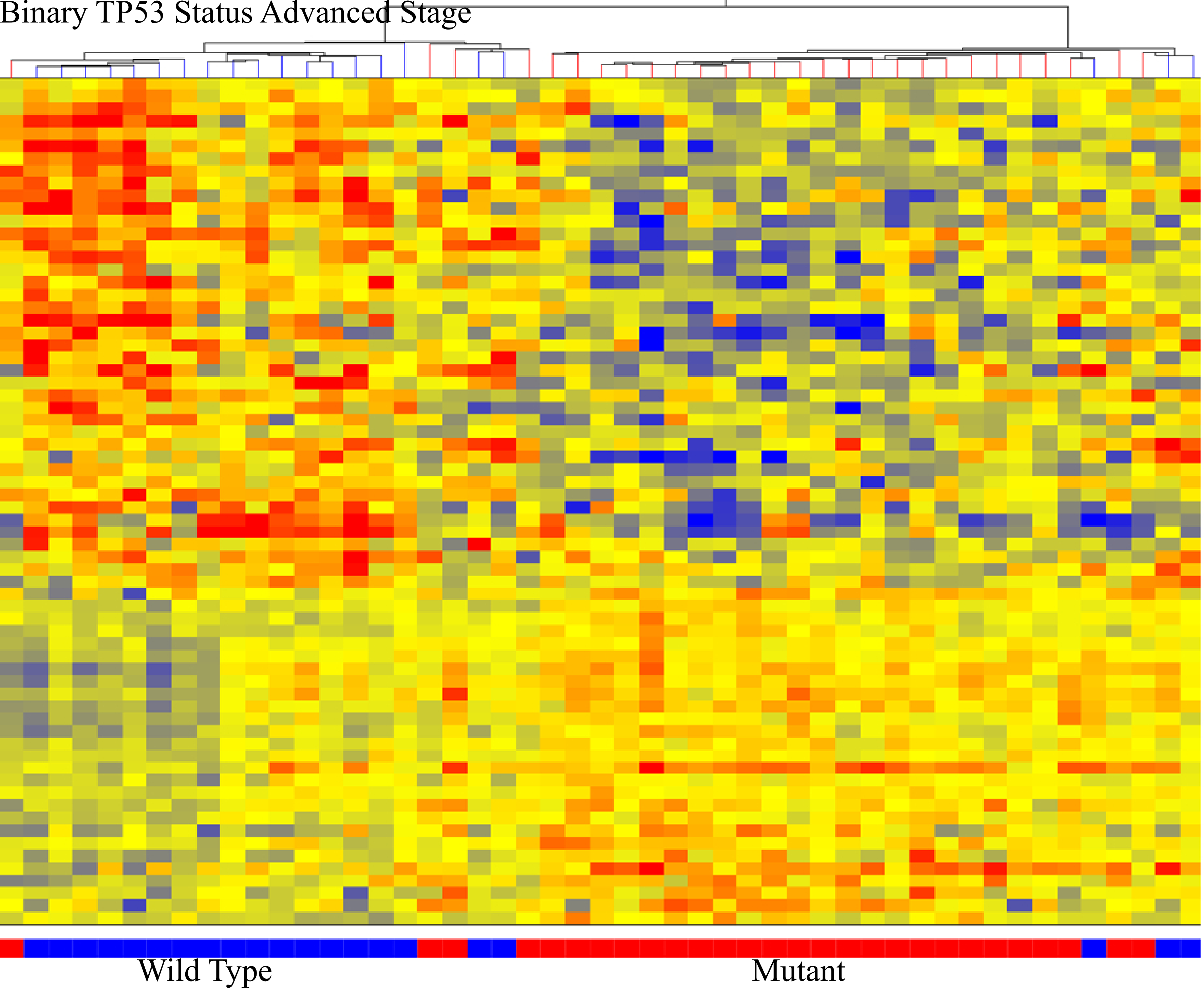

Binary TP53 Status Advanced Stage
Wild Type
Mutant

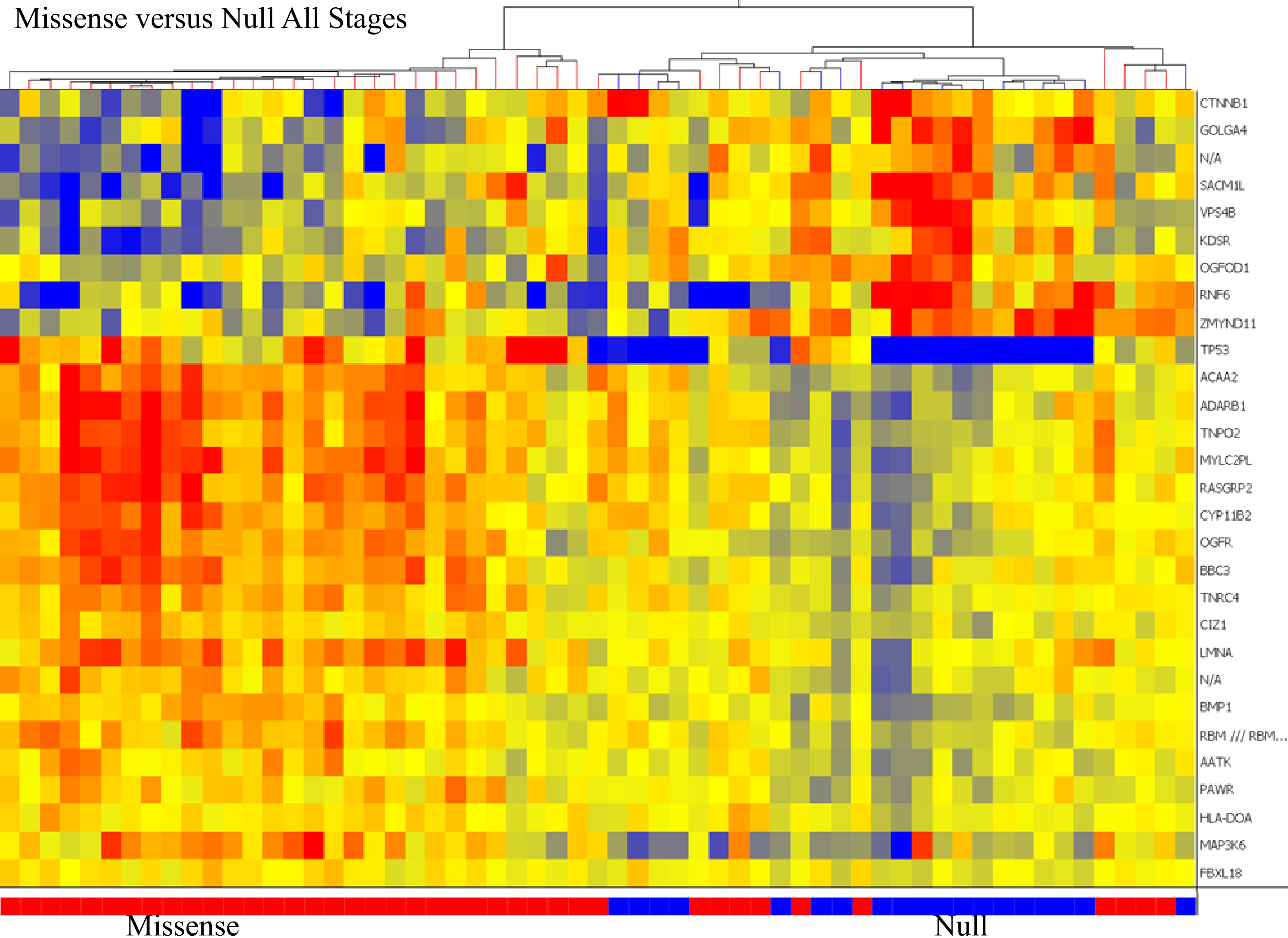

Missense versus Null All Stages
CTNNB1
GOLGA4
N/A
SACM1L
VPS4B
KDSR
OGFOD1
RNF6
ZMYND11
TP53
ACAA2
ADARB1
TNPO2
MYLC2PL
RASGRP2
CYP11B2
OGFR
BBC3
TNRC4
CIZ1
LMNA
N/A
BMP1
RBM /// RBM...
AATK
PAWR
HLA-DOA
MAP3K6
FBXL18
Missense
Null

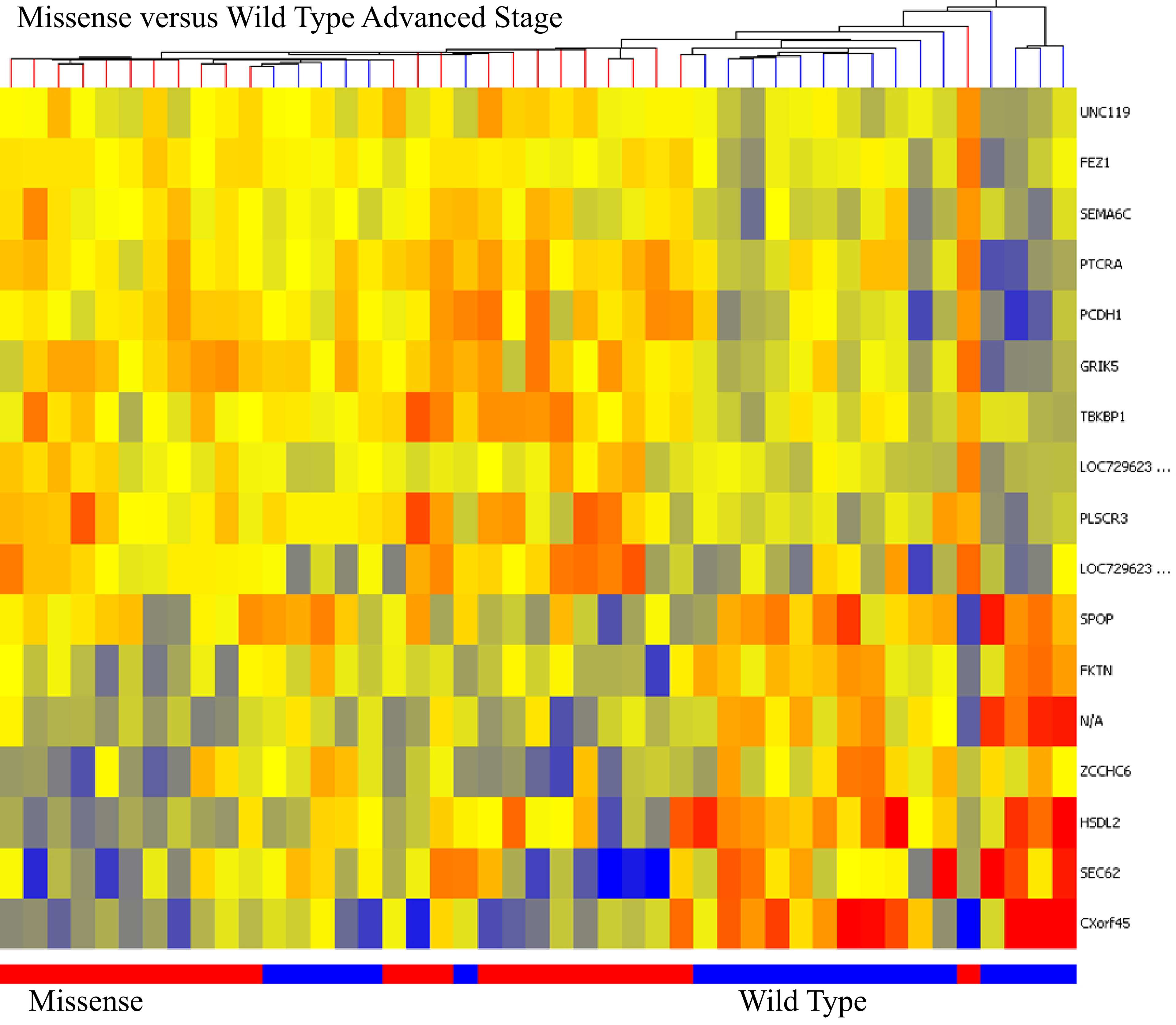

Missense versus Wild Type Advanced Stage
UNC119
FEZ1
SEMA6C
PTCRA
PCDH1
GRIK5
TBKBP1
LOC729623 ...
PLSCR3
LOC729623 ...
SPOP
FKTN
N/A
ZCCHC6
HSDL2
SEC62
CXorf45
Missense
Wild Type

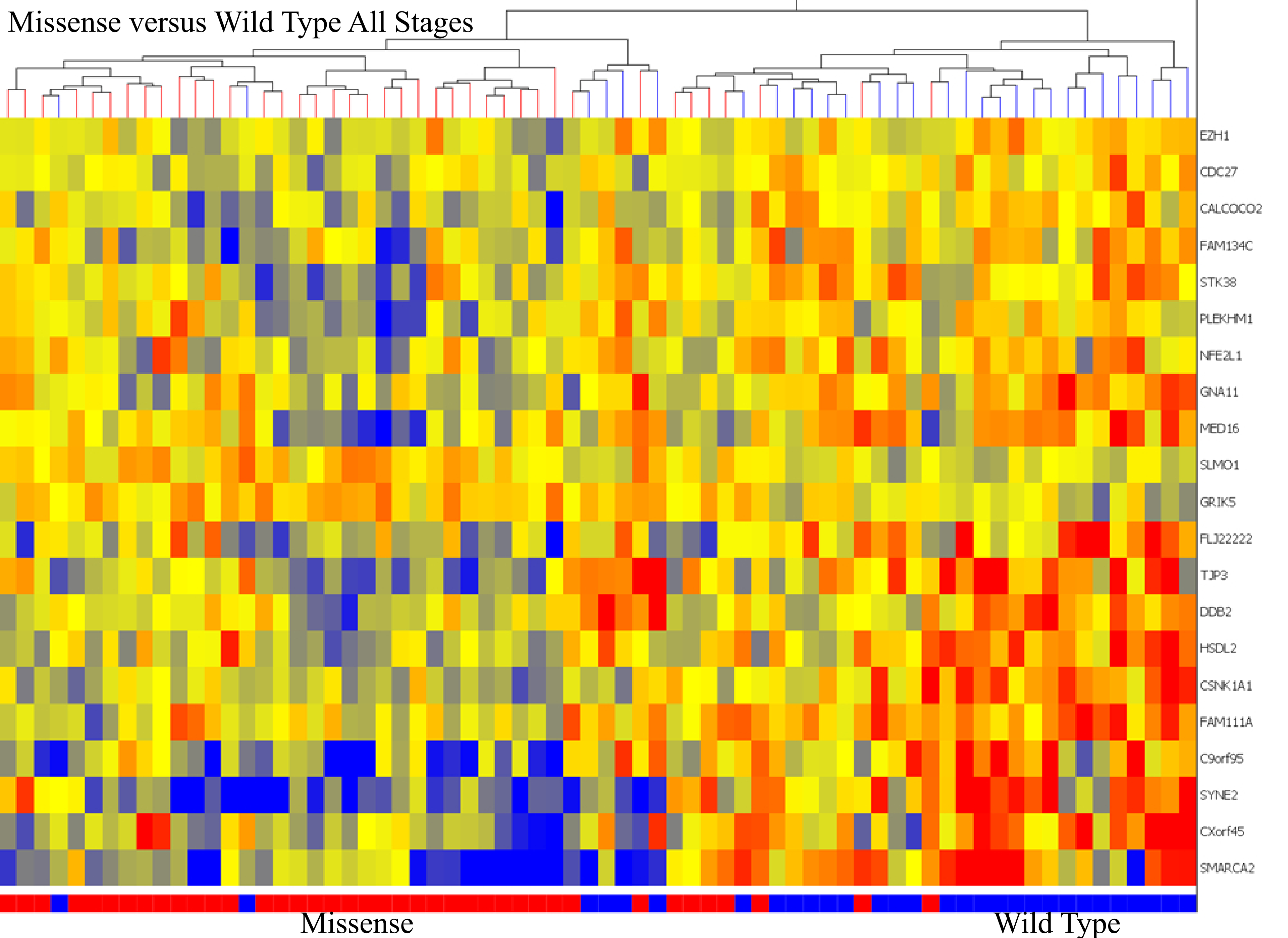
Missense versus Wild Type All Stages
EZH1
CDC27
CALCOCO2
FAM134C
STK38
PLEKHM1
NFE2L1
GNA11
MED16
SLMO1
GRIK5
FLJ22222
TJP3
DDB2
HSDL2
CSNK1A1
FAM111A
C9orf95
SYNE2
CXorf45
SMARCA2
Missense
Wild Type

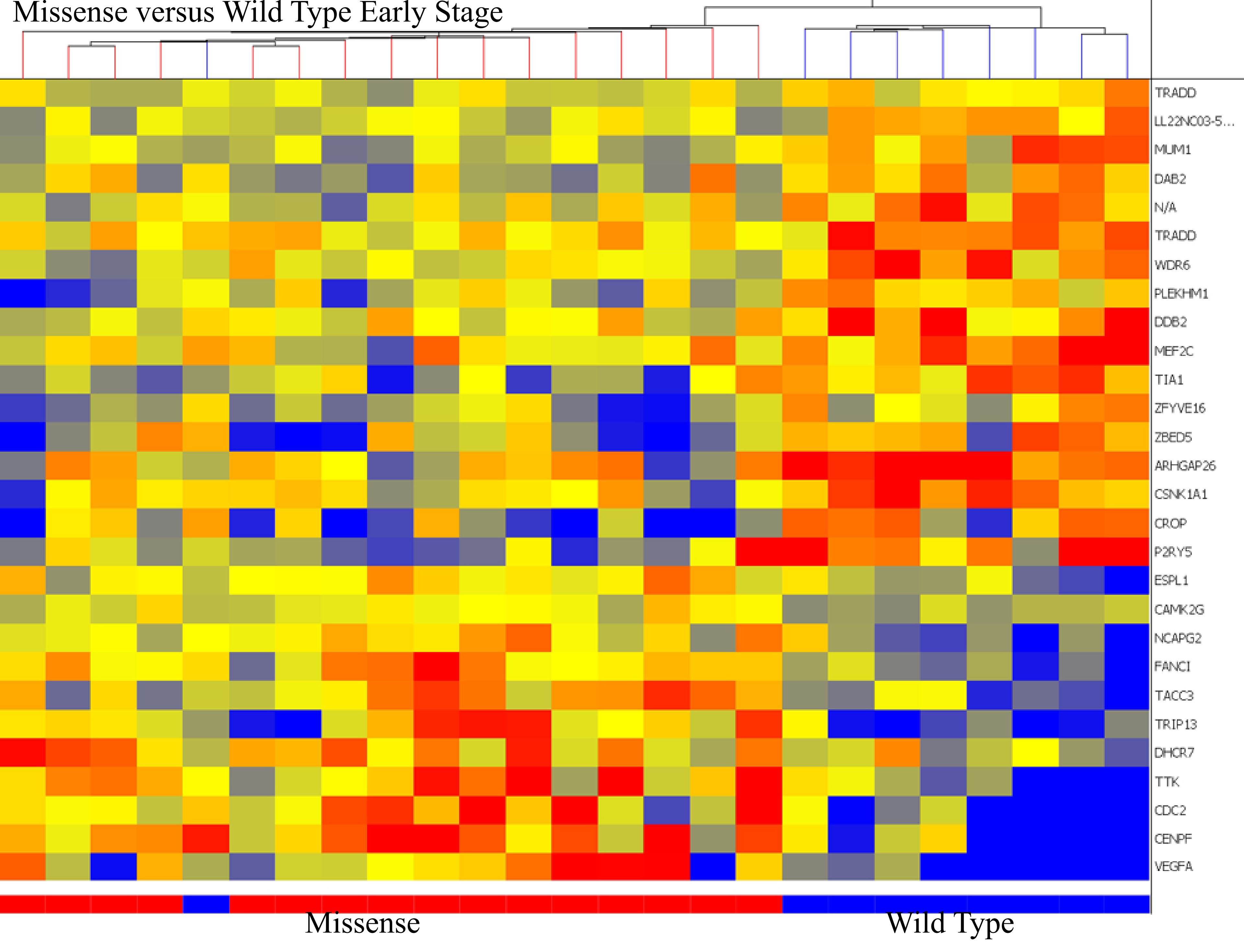
Missense versus Wild Type Early Stage
TRADD
LL22NC03-5...
MUM1
DAB2
N/A
TRADD
WDR6
PLEKHM1
DDB2
MEF2C
TIA1
ZFYVE16
ZBED5
ARHGAP26
CSNK1A1
CROP
P2RY5
ESPL1
CAMK2G
NCAPG2
FANCI
TACC3
TRIP13
DHCR7
TTK
CDC2
CENPF
VEGFA
Missense
Wild Type
