# Supplementary material for: Expression signatures of TP53 mutations in serous ovarian cancers
Source: BMC Cancer. 2010 May 26;10:237. doi: 10.1186/1471-2407-10-237 (PMC2893110; doi:10.1186/1471-2407-10-237)
Supplement: Additional File 4 — Affymetrix DASL Correlations. The comparison of expression values for selected genes for a subset of cancers between Affymetrix data derived from frozen material and DASL data derived from the same cancers that were formalin-fixed and paraffin embedded. [file 1471-2407-10-237-S4.PDF]

Correlation between Illumina DASL and Affymetrix gene expression values for 38 advanced stage cancers.

Gene	Pearson Correlation	P Value
TJP3	0.32	**0.05**
DDB2	0.51	**0.0011**
AARSD1	0.48	**0.0023**
PLKHM1	0.32	**0.05**
CXorf45	0.25	0.14
EZH1	0.16	0.32
FAM111A	-0.03	0.84
MED16	0.32	**0.05**
CSNK1A1	-0.13	0.45

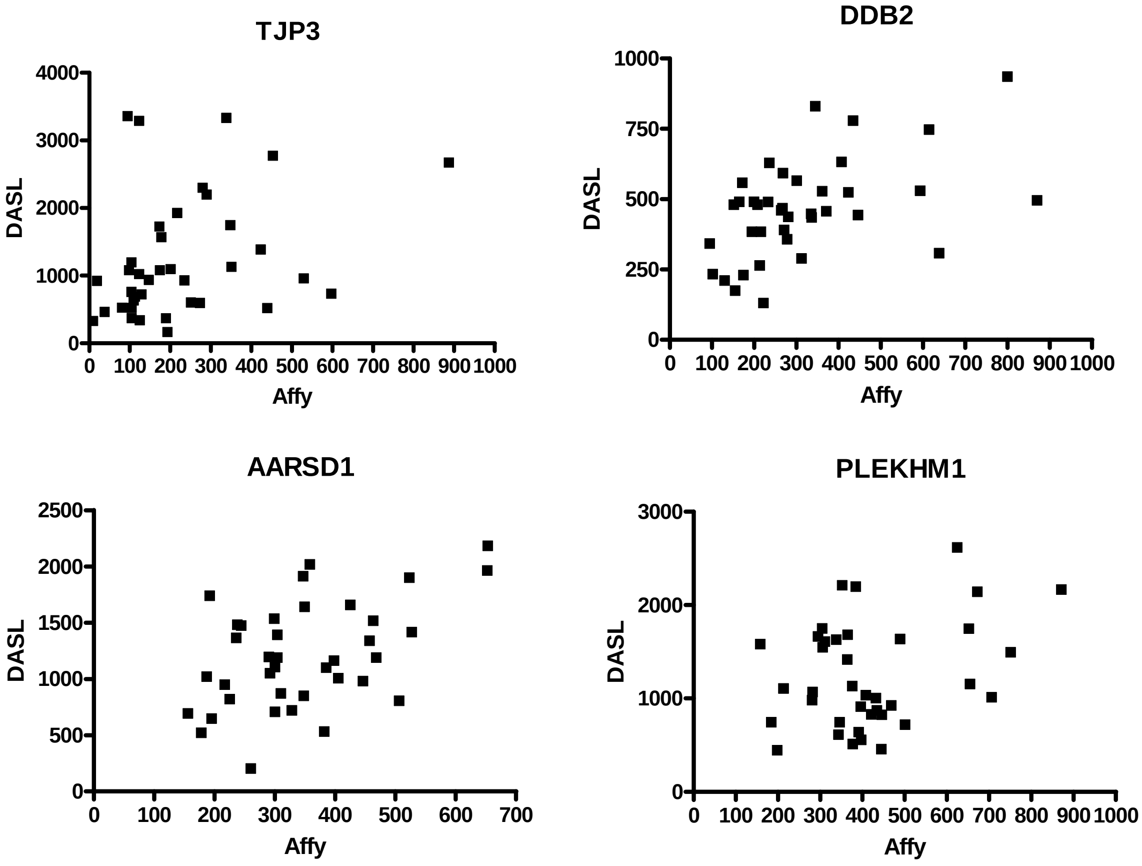

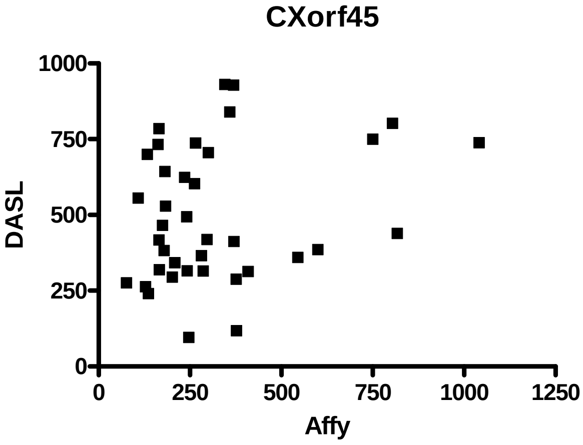
CXorf45
DASL
0
250
500
750
1000
Affy
0
250
500
750
1000
1250

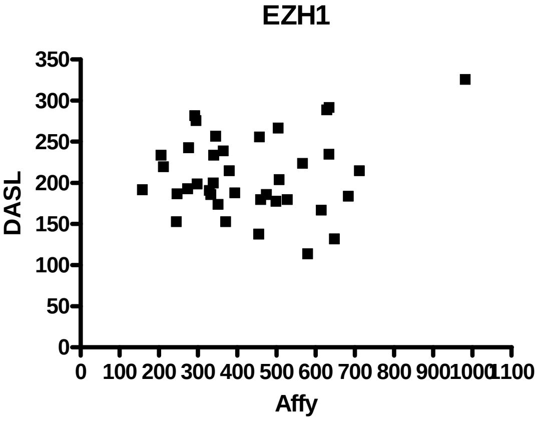
EZH1
DASL
0
50
100
150
200
250
300
350
Affy
0
100
200
300
400
500
600
700
800
900
1000
1100

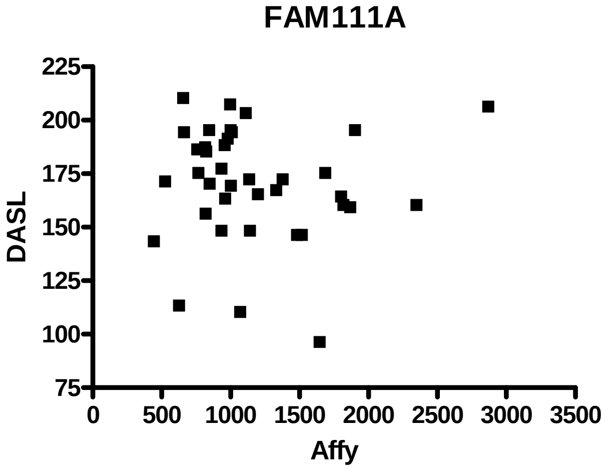
FAM111A
DASL
75
100
125
150
175
200
225
Affy
0
500
1000
1500
2000
2500
3000
3500

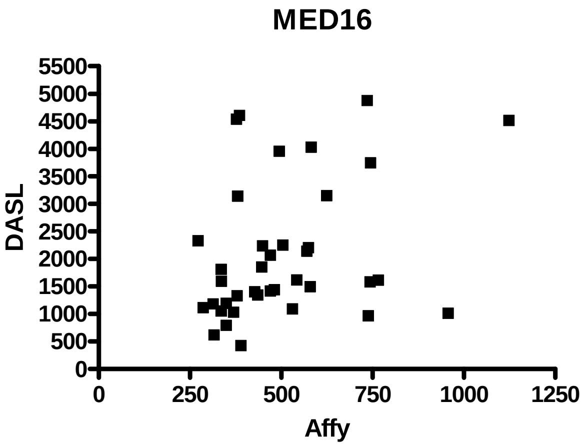
MED16
DASL
0
500
1000
1500
2000
2500
3000
3500
4000
4500
5000
5500
Affy
0
250
500
750
1000
1250

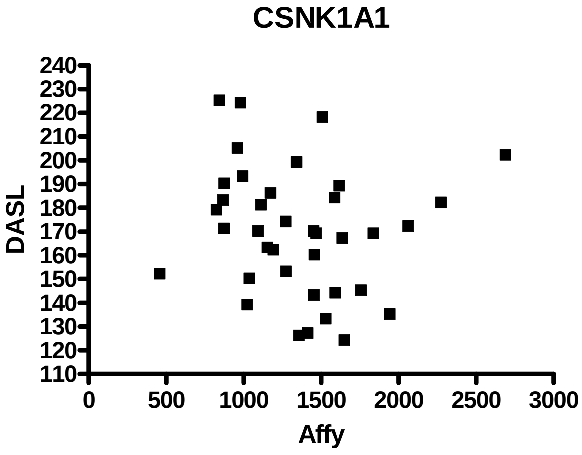
CSNK1A1
DASL
110
120
130
140
150
160
170
180
190
200
210
220
230
240
Affy
0
500
1000
1500
2000
2500
3000
